# Large Scale Production and Downstream Processing of Labyrinthopeptins from the Actinobacterium *Actinomadura namibiensis*

**DOI:** 10.3390/bioengineering5020042

**Published:** 2018-06-05

**Authors:** Zeljka Rupcic, Stephan Hüttel, Steffen Bernecker, Sae Kanaki, Marc Stadler

**Affiliations:** 1 Department Microbial Drugs, Helmholtz Centre for Infection Research GmbH, Inhoffenstraße 7, 38124 Braunschweig, Germany; zeljka.rupcic@helmholtz-hzi.de (Z.R.); steffen.bernecker@helmholtz-hzi.de (S.B.); 2 German Centre for Infection Research (DZIF), partner site Hannover-Braunschweig, 38124 Braunschweig, Germany; 3 Toyama Prefectural University, 5180 Kurokawa Imizu-shi, Toyama 939-0398, Japan; t416010@st.pu-toyama.ac.jp

**Keywords:** anti-viral agents, bioprocess, central nervous system, lantibiotics, optimization production, reversed phase-high performance liquid chromatography, scale-up

## Abstract

A method was established for the production of 1.2-fold and 4.2-fold increased amounts of the antiviral and central nervous system-active lantipeptides, labyrinthopeptins A1 and A2, respectively, isolated from the actinobacterium *Actinomadura namibiensis*, to enable production in gram scale. We then performed in vivo characterization of this promising compound class. The labyrinthopeptins A1 and A2 have similar chemical structures and physical properties but differ drastically in their bioactivities. Therefore, large quantities of highly pure material are required for pharmacological studies. An effective methodology was established for the first time for their production in bioreactors, their separation involving gel permeation chromatography on LH20 material, followed by reversed phase-high performance liquid chromatography. With an optimized methodology, 580 mg of labyrinthopeptin A1 and 510 mg of labyrinthopeptin A2 were quantitatively isolated with recovery rates of 72.5% and 42.3% from 7.5 L of culture broth, respectively. However, the fermentation that had already resulted in maximum yields of over 100 mg/L of both target molecules after 300 h in a 10-L scale bioreactor, still requires further optimisation.

## 1. Introduction

Several groups of Gram-positive bacteria, such as actinobacteria, lactobacilli, and staphylococci, are able to ribosomally biosynthesize oligopeptides from 18–38 amino acids, called lantibiotics or lantipeptides. Their common structural feature is the presence of noncanonical thioether amino acids like lanthionine and/or methyllanthionine (MeLan) [1,2]. The food preservative nisin is the most important commercial representative of this compound family [3]. According to their biosynthetic pathways, lantibiotics are classified into three major types: Class I lantibiotics are modified by separate dehydratases (LanB) and cyclases (LanC), whereas class II lantibiotics are dehydrated and cyclized by a single LanM-type enzyme. Unlike classes I and II, class III lantibiotics have little or no antibiotic activity; rather, they provide alternative physiological functions [1]. An example of class I lantibiotics, aside from the aforementioned nisin, are the recently characterized pinensins, which represent the first antifungal lantibiotics isolated from a Gram-negative bacterium [4]. Pseudomycoicidin, an antibacterial representative of class II lantibiotics, was isolated from a Gram-positive bacterium [5]. From actinobacteria, only four peptides have previously been described and structurally characterized as type III lantipeptides, although comparative genomic studies have revealed that homologous gene clusters are abundant in other strains of this bacterial group [6]. An example of lantibiotics in phase II clinical trials is duramycin (moli1901), which is being developed for the treatment of cystic fibrosis as it increases chloride transport in the airway epithelium [7]. 

**Figure 1:**
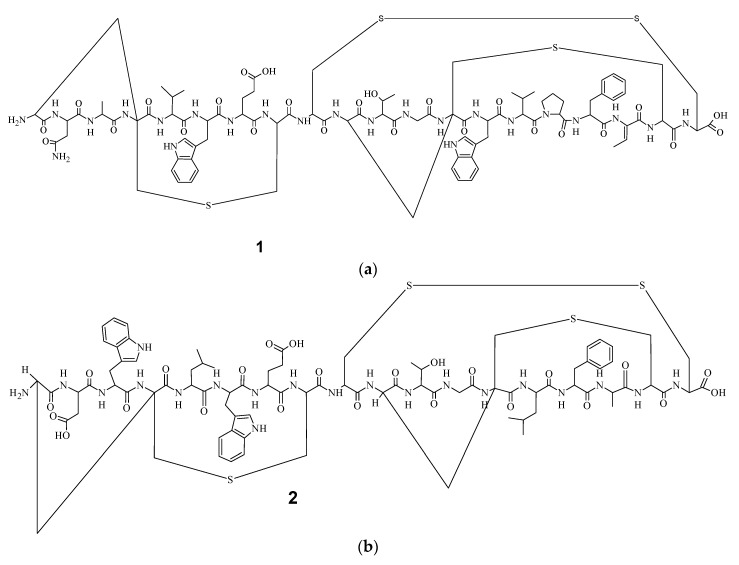
Chemical structures of (**a**) labyrinthopeptin A1 (**1**) and (**b**) labyrinthopeptin A2 (**2**).

**Figure 2:**
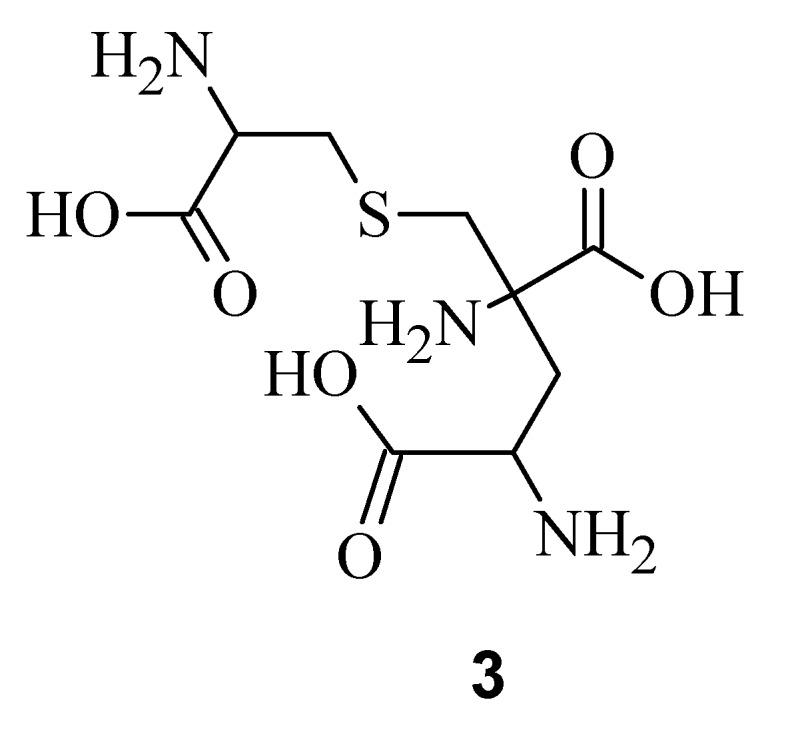
Chemical structure of labionin (**3**).

The current study was dedicated to the labyrinthopeptins, which were first isolated from the desert actinobacterium *Actinomadura namibiensis* within Aventis in 1988 [8], but their structure was only determined recently [9]. Electrospray Ionization Fourier Transform Ion Cyclotron Resonance (EIFTCR) mass spectrometry of its crude extract showed three labyrinthopeptin derivatives: labyrinthopeptin A1 (**1**) (Figure 1a), labyrinthopeptin A2 (**2**) (Figure 1b), and labyrinthopeptin A3 (**3**), a degradation product of **1** [10]. All three derivatives possess a post translationally modified triamino acid labionin (Figure 2) [10,11].

Because of their biological activities, labyrinthopeptins have the potential to become lead compounds for drug development in two different indications. For instance, **2** displayed an activity in a spared nerve injury mouse model of neuropathic pain, whereas **1** exhibited in vitro antiviral effects against Human Immunodeficiency Virus (HIV) and Herpes Simplex Virus (HSV) at sub-micromolar concentrations [10]. Compound **1** also showed synergistic effects with standard antiretroviral drugs and the absence of a PBMC inflammatory response. Since they did not interact with vaginal lactobacilli, labyrinthopeptin A1 (**1**) is an ideal candidate for the treatment of sexually transmitted viruses, as well as for development as a broad spectrum antiviral agent [12]. The latter hypothesis was confirmed in a study where both labyrinthopeptins showed activity against human respiratory syncytial virus (hRSV) subtype A and B [13]. The mechanism of action for this antiviral activity is still not completely understood, but the activity may be related to the blockage of viral entry by interacting with the viral envelope and preventing cell-to-cell transmission [12].

Labyrinthopeptins were produced by biotechnological means yielding 6.2 mg/L of **2**, obtained from a shake flask batch cultivation in 10 L scale [10]. Another study by Krawczyk et al. [14] reported 90 mg/L for **1** and 39 mg/mL for **2** obtained from the wild type strain of *Actinomadura namibiensis*. However, details on the purity of the compounds were not provided in that study. In the current study, for the first time, we describe a scalable batch-process using a 10 L bioreactor and *Actinomadura namibiensis* strain DSM 6313 as the producer organism, which led to a 1.2-fold increase in the yield of **1**, and a 4.2-fold increase in the yield of **2** compared to the data in the literature [14]. Moreover, we outline an optimized downstream processing procedure that ensures a quantitative supply of **1** and **2** with sufficient purity for further pre-clinical studies.

## 2. Materials and Methods

### 2.1. General Experimental Procedure

All HPLC analyses were performed on an Agilent 1200 Series instrument (Agilent, Santa Clara, CA, USA), equipped with degasser, binary Pump SL (Agilent Technologies 1260 Infinity, Agilent Technologies, Santa Clara, CA, USA), autosampler and a combined diode array detector/electron light scattering detector Corona Ultra RS (Dionex, Sannyvale, CA, USA), using the conditions described by Beckmann et al. [15]. 

High performance liquid chromatography-electrospray ionization mass spectrometry (HPLC-ESI-MS) spectra were recorded with an Agilent 1200 series HPLC system with Acquity UPLC BEH C18 column (2.1 × 50 mm, 1.7 µm) from Waters (Eschborn, Germany), coupled to an ion trap mass spectrometer, amZon™ (Bruker, Bremen, Germany) (scan range 100 to 2000 *m/z*, capillary voltage 4000 V, dry temperature 250 °C).

Ultra High Resolution-Time of Flight (UHR-TOF-MS) data were obtained using an Ultimate 3000RS Thermo Scientific™ Dionex™ (Waltham, MA, USA) instrument equipped with a Kinetex C18 column (150 × 2.1 mm, 1.7 µm) from Phenomenex (Torrance, CA, USA) as stationary phase. The HPLC system was coupled to a mass spectrometer (maXis HD™, Bruker, Bremen, Germany), with a scan range from 250 to 2500 *m/z*, a set collision energy of 8.0 eV, capillary voltage 4500 V, nebulizer 4.0 bar, and the dry heater set to 200 °C. 

Chemicals and solvents were obtained from AppliChem GmbH (Darmstadt, Germany), Avantor Performance Materials (Deventor, The Netherlands), Carl Roth GmbH & Co. KG (Karlsruhe, Germany), and Merck KGaA (Darmstadt, Germany) in analytical and HPLC grade.

### 2.2. Fermentation in a 10 Liter Scale Bioreactor

A seed culture of *Actinomadura namibiensis* DSM 6313 was prepared in two steps. An aliquot of 1.8 mL from a cryo culture in 10% glycerol was inoculated in 100 mL of the production medium, starch-glucose-glycerol (SGG) consisting of 10 g/L starch (Cargill, Sas van Gent, The Netherlands), 2 g/L yeast extract (Ohly®KAT GmbH, Hamburg, Germany), 10 g/L glucose (Cerestar, Neuilly-Sur-Seine, France), 10 g/L glycerol (Roth, Karlsruhe, Germany), 2.5 g/L corn steep powder (Sigma-Aldrich, Darmstadt, Germany), 2 g/L peptone (Markor, Carlstadt, NJ, USA), 1 g/L sodium chloride (NaCl), 3 g/L calcium carbonate (CaCO_3_), both from Roth in tap water at pH 7.0 before sterilization, in a 250 mL Erlenmeyer flask and incubated on a rotary shaker for 72 h at 30 °C and 160 rpm. For the preparation of a secondary seed culture, 20 mL of each of the first inoculum were added to 1000 mL sterile Erlenmeyer flasks with a 400 mL working volume of SGG medium, and then incubated for 48 h at 30 °C and 160 rpm.

**Figure 3:**
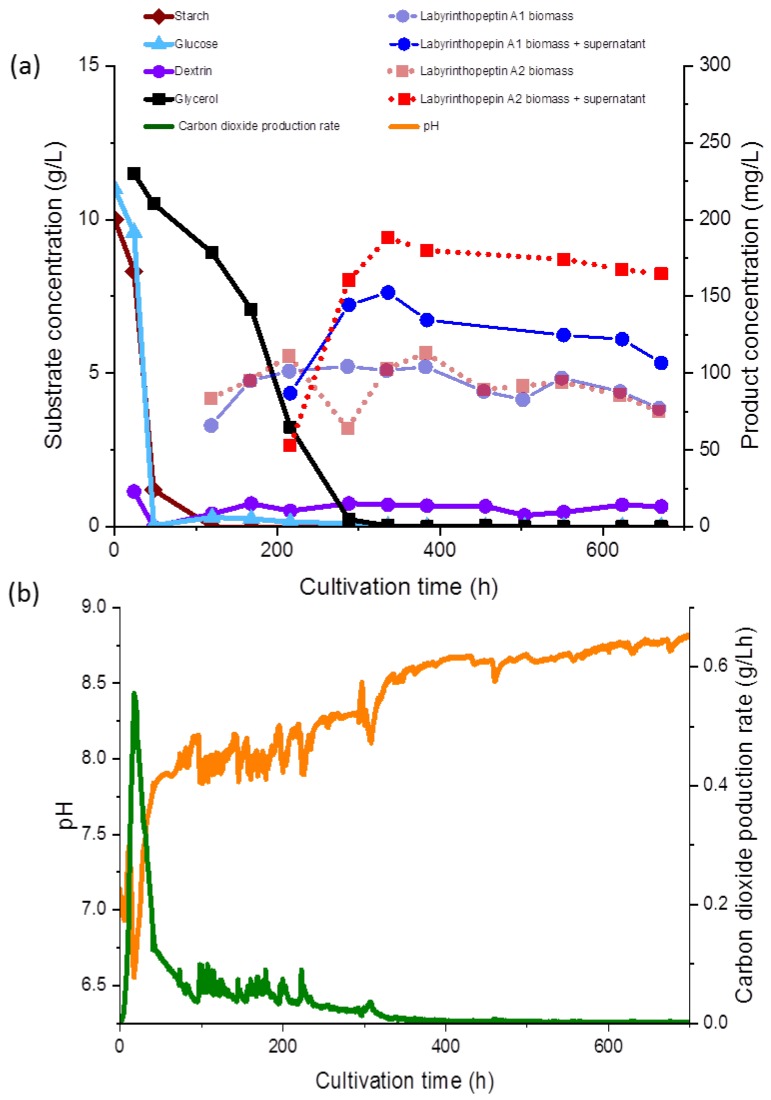
(**a**) Correlation between product concentration (mg/L) and substrate concentrations (g/L); (**b**) carbon dioxide production (CPR; g/Lh) and pH determined during regular time intervals in a 10 L batch fermentation. Product concentration is expressed as a cumulative value for the biomass and the supernatant together (dashed lines) and for the biomass only (solid lines).

The batch cultivation was performed in a 10 L steel reactor (xCUBIO in situ, bbi biotech, Berlin, Germany). The system was equipped with an aseptic sampling system probe, and analyzers for exhaust O_2_ and CO_2_, pH, pO_2_ (clarc) and temperature, level and foam sensors, and three rushton impellers for agitation. The pH was adjusted prior to 7.2 fermentation by addition of H_2_SO_4_ or KOH, and was not maintained during the fermentation (Figure 3b). The gas flow rate was maintained at 0.25 vvm (2.5 nL/min), and the temperature was set to 30 °C. Stirring was set to a minimum of 100 rpm and automatically increased to maintain a pO_2_ of 30%. Batch fermentation was started with a cultivation volume of 9.5 L of SGG medium. A 5% inoculum was added and the fermentation was continued for 672 h.

### 2.3. Determination of Substrate and Product Concentrations during the Fermentation

Glucose, glycerol, and dextrin concentrations were determined as described previously [14] using an Agilent 1260 series HPLC and a Phenomenex (Aschaffenburg, Germany) REZEX ROA-Organic Acid H+ (8%) column (300 × 7.8 mm × 8 µm) at 65 °C with a refractive index detector. Separation occurred under isocratic conditions of 0.05 mM H_2_SO_4_ for 45 min (Figure 3a). The carbon dioxide production rate (CPR) was automatically monitored and the time course is shown in Figure 3b.

The product concentration was determined separately for the biomass and the supernatant. For this purpose, samples (2 × 10 mL) were obtained at regular intervals. To the first sample, 1% (*v*/*v*) Amberlite XAD™-16N adsorber resin (Sigma-Aldrich, St. Louis, MO, USA) was added and centrifuged for 20 min at 5000 rpm. Thereafter, the supernatant was discarded, 12 mL of a mixture of acetone, methanol (MeOH), and water (1:3:2) was added, sonicated for 30 min at 40 °C, and centrifuged for 20 min at 5000 rpm. The organic solvent was evaporated *in vacuo* and the aqueous residue was freeze-dried.

The second sample was treated as described above, but without the Amberlite XAD™-16N adsorber resin, to determine the product concentration in pellet for only. Both extracts were reconstituted in the MeOH and H_2_O mixture (50:50, *v*/*v*), centrifuged for 5 min at 14,000 rpm and the supernatants were injected into a Dionex Ultimate 3000 (Thermo Scientific, Waltham, MA, USA) HPLC system, using a Nucleodur Phenyl Hexyl column (1.8 µm; 100 × 2 mm, Macherey Nagel, Düren, Germany) as stationary phase, equipped with a pre-column consisting of the same material (1.8 µm; 4 × 2 mm). The mobile phase was composed of solvent A, H_2_O + 0.1% formic acid (FA), and solvent B, acetonitrile (ACN + 0.1% FA). A gradient was run from 30% B for 1 min, followed by a linear increase to 50% B over 10 min, which was afterward increased to 100% B in 1 min, and then maintained under isocratic conditions at 100% for 5 min, at a flow rate of 0.3 mL/min and ultraviolet (UV) detection at 280 nm. The product concentrations were determined via a calibration curve using five defined standard solutions, as displayed in Figure 3a in relation to the substrate concentration.

### 2.4. Downstream Processing and Isolation of Labyrinthopeptins

The culture broth (7.5 L) was centrifuged at 9000 rpm (Sorvall^®^ RC5B centrifuge, DuPont Instruments, Wilmington, DE, USA) for 30 min to separate the biomass (975 mL) from the supernatant. The resulting biomass cake (529 g) was defatted with an equal amount of *n*-heptane, extracted two times with a mixture of 7.5 L acetone, MeOH, and H_2_O (1:3:2) for 1 h in an ultraconic bath Sonorex Digital 10P (Bandelin, Berlin, Germany). The organic solvent was evaporated *in vacuo* and the remaining aqueous residue was freeze-dried to avoid losses due to the extensive foaming of the extract at lower pressure. We obtained 8.8 g of crude product.

The culture broth was incubated with 1% (*v*/*v*) of Amberlite XAD™-16N adsorber resin under stirring for 1 h. The resin was removed by filtration and subsequently extracted with 1 L of solvent mixture, according to the same protocol described above, to obtain 1.7 g of crude extract.

Both the mycelial and the supernatant extracts were dissolved in a mixture of MeOH and H_2_O (50:50, *v*/*v*), centrifuged for 15 min at 5000 rpm, and purified via gel permeation chromatography (GPC) on Sephadex® LH 20 material (Pharmacia Fine Chemicals, Inc., New York, NY, USA). A medium pressure liquid chromatography (MPLC) device was used for the GPC, equipped with Minipuls S3 pump (Gilson, Inc.; Middleton, WI, USA), a B 687 mixing device (Büchi, Flawil, Switzerland), a 2138 UVICORDS UV detector set to 220 nm (LKB, Bromma, Stockholm, Sweden), and a REC 102 writer (Pharmacia Biotech Inc., Piscataway, NJ, USA).

The mobile phase was composed of MeOH:H_2_O (50:50, *v*/*v*), the flow rate of 8 mL/min was applied, with an overall solvent consumption of 7.7 L to create two fractions at the retention times of 108 min and 124 min. The fractions were subsequently checked by HPLC-ESI-MS to determine peptide co-elution and therefore, both were pooled together. A total of 5 g and 0.8 g of intermediate product was obtained after GPC, which was further purified via the preparative HPLC. For this purpose, a Gilson GX270 Series HPLC system was used with a Nucleodur Phenyl hexyl column (5 µm; 150 × 40 mm; Macherey-Nagel, Düren, Germany), solvent A with H_2_O + 0.1% FA, and solvent B with ACN + 0.1% FA. Gradient elution was performed as follows: 5 min isocratic on 30% of B, followed by a linear increase to 80% of B over 30 min, then an increase of B to 100% over 5 min, and maintaining at 100% of B for the next 5 min. A flow rate of 30 mL/min was used and detection was performed at 254 nm. Fractions were collected according to the observed peaks and were submitted to UHR-TOF-MS. For chromatograms, see Supplementary Figures S1–S7. Finally, 420 mg and 160 mg of **1** were isolated from the biomass and supernatant, respectively, with 99.34% purity. From **2**, 380 mg were isolated from the biomass crude extract, whereas 140 mg were obtained from the supernatant crude extract with 99.13% purity. For their isolation at the preparative scale, 26 L of ACN and 22.7 L of H_2_O were consumed, whereas 42 h were required for obtaining the yields. The mass balance for the major unit operations of the downstream processing is displayed in Table 1 and Table 2 for the biomass and the supernatant, respectively, whereas recovery of the isolation process is provided in Table 3.

## 3. Results and Discussion

According to unpublished experiments, **1** and **2** differ drastically in their bioactivity. To enable further in vivo drug development studies, substantial amounts of the compounds are required as well as high purity peptides. Since cost-efficient synthesis is not possible, their production can only be attained by means of biotechnological production. 

Conversely, heterologous production of these ribosomally synthesized and posttranslationally modified peptides was reported previously [14]. Since attempts to establish a suitable genetic system in the wild-type producer *Actinomadura namibiensis* failed, a heterologous *Streptomyces* host was used instead. A general obstacle to the heterologous expression in *S. lividans* is the undesired production of labyrinthopeptin variants with additional N-terminal amino acids. The authors reported using different constructs, pLab_SG6, for the exclusive generation of **2**, resulting in a yield of 14 mg/L, whereas **1** was generated by the pLab_SG6 construct (86 mg/L). The titers obtained were in the range of the wild-type *Actinomadura namibiensis*, at 90 and 39 mg/L for **1** and **2**, respectively [14]. However, this process was never scaled up to attain quantitative amounts of the desired products. These constructs may not provide a path forward to transfer production into pilot scale bioreactors, despite the fact that the considerably increased yields compared to those initially reported yields from shake flasks (6.2 mg/L for **2**) [10]. 

During fermentation, various observations were recorded regarding the correlations of the substrate consumption, which was monitored during the course of our study for the first time during production of the labyrinthopeptins, and metabolite production. All carbon sources used in the media, except for glycerol, were depleted within 48 to 96 h. The starch was metabolized at the initiation of the fermentation and consequently, after the starch was hydrolyzed, an increased concentration of dextrin was measured (<1.0 g/L). Free glucose was only detected at the beginning of the fermentation at a concentration of 0.05 g/L, until 48 h after inoculation, whereas glycerol decreased gradually until 336 h (Figure 3a). The lowest measured pH value over the fermentation time was 6.93 (Figure 3b), but the pH showed a growing tendency toward the end of the fermentation. The sampling started 120 h after the inoculation and 65 mg/L and 83 mg/L of labyrinthopeptins from A1 (**1**) and A2 (**2**) were calculated, respectively, although we assumed from preliminary shake flask experiments the production started shortly before the first measurement (data not shown). Nevertheless, preliminary experiments showed that the production occurred after sugars and their oligomers had been consumed and the strain entered a phase of limited respiratory activity as illustrated by the CPR where glycerol was then used as a carbon source (Figure 3a). 

At the end of the fermentation, the concentrations of **1** and **2** were estimated to be approximately 106 and 165 mg/L, respectively. Interestingly, compound **1** was predominant in terms of biomass throughout the entire cultivation, whereas **2** was equally represented in both the supernatant and the biomass. After 672 h of fermentation, increased concentrations of **2** in the supernatant were observed (Figure 3a,b). An anomaly in the product concentration was observed after 500 h of fermentation, where a decreased concentration in the product obtained from the supernatant was measured. This measuring point was eliminated from the graph in Figure 3a. We depicted the fermentation from which the product was ultimately isolated, but since the product titers remained stable, terminating the fermentation much earlier in the future would be possible, after about 300 h. Several other options to further increase the titers and reduce the fermentation time, such as by increasing the inoculum and conduction of fed-batch experiments, are presently being planned, and those will commence in parallel to the transfer of the process to 70- and 250-L scales. 

After preparative separation, only about 50% of the estimated value of compound **2** was recovered by chromatography, resulting in 510 mg of isolated compound. Nevertheless, this yield constitutes a 4.2-fold increase compared to the data found in the literature [14]. A titer of 106 mg/L was obtained for compound **1** with a recovery rate of 72.5%, resulting in the isolation of 580 mg of the compound (Table 3). As compared to the data in the literature, this yield constitutes a 1.2-fold increase [14]. However, this study did not include experiments on the quantitative isolation of **1** and no recovery rates are provided. In comparison to the titer for **2**, we obtained a considerably lower titer for **1**; however, larger amounts of **1** were quantitatively separated from the crude product (Table 1 and Table 2).

Since the compounds have a high molecular weight and as large sample amounts had to be handled, the downstream processing was accomplished with the combination of a GPC, as a pre-purification step, with the Sephadex^®^ LH 20 material, and finally by the purification by preparative HPLC. As expected, the labyrinthopeptins were eluted concurrently, but separating the majority of the co-metabolites and media constituents from the mixture of **1** and **2** was possible. For the final purification of the two target compounds, preparative chromatography had to be used.

Our work clearly demonstrates a substantial increase in the production of labyrinthopeptins and provides a straightforward approach to downstream processing, even for future attempts to further scale-up the process to the pilot scale. From our hindsight of the bioprocess development, different media and different ratios of media components are concurrently being tested to achieve even higher titers in the future, and the process is ready for transfer to the pilot scale. 

## 4. Conclusions

A method for the biotechnological production, together with a new method for providing sustainable accessibility of the two structurally similar lantibiotics was established in the current study. By improving the isolation/separation conditions, labyrinthopeptins A1 and A2 were obtained at final recovery rates of 72.5% and 42.3%, respectively, and purities of over 99% as estimated by HPLC-UV were attained for both metabolites. To fulfil the requirements for future evaluation of the compound class, involving both, animal studies, and the formulation experiments, gram amounts of labyrinthopeptins are needed. The results presented here provide a path forward, since the compounds were obtained at very high purity, and both the GPC method and the final HPLC separation step can be scaled up in a straightforward manner. A concurrent extensive optimization of culture media and fermentation parameters, in order to further increase the yields and recovery rates will be necessary in any case, in order to attain favorable costs of goods and will be the subject of our further research.

## Figures and Tables

**Table 1:** Mass balance of the input and the output of major unit operations in the biomass downstream processing.

**Process Step**	**Input (g)**	**Output A1**		
		**A1 (g)**	**Recovery A1 (%)**	**Loss A1 (%)**
Crude extract	8.8	0.57	-	0
GPC (3 runs) ^1^	5.0	0.45	78.9	21.1
Prep. HPLC (25 runs) ^1^	0.8	0.42	93.3	6.7
**Process Step**	**Input (g)**	**Output A2**		
		**A2 (g)**	**Recovery A2 (%)**	**Loss A2 (%)**
Crude extract	8.8	0.55	-	0
GPC (3 runs) ^1^	5.0	0.45	81.8	18.2
Prep. HPLC (25 runs) ^1^	0.8	0.38	84.4	15.6

^1^ For the complete downstream process of the biomass.

**Table 2:** Mass balance of the input and the output of major unit operations in the supernatant downstream processing.

**Process Step**	**Input (g)**	**Output A1**		
		**A1 (g)**	**Recovery A1 (%)**	**Loss A1 (%)**
Crude extract	1.7	0.23	-	0
GPC (1 run) ^2^	0.8	0.18	78.3	21.7
Prep. HPLC (5 runs) ^2^	0.3	0.16	88.9	11.1
**Process Step**	**Input (g)**	**Output A2**		
		**A2 (g)**	**Recovery A2 (%)**	**Loss A2 (%)**
Crude extract	1.7	0.68	-	0
GPC (1 run) ^2^	0.8	0.28	41.2	58.8
Prep. HPLC (5 runs) ^2^	0.3	0.14	42.4	57.6

^2^ For the complete downstream process of the supernatant.

**Table 3:** Overall-recovery of labyrinthopeptins A1 and A2 from the isolation and separation process.

Compounds	Amount Estimated (g)	Amount Isolated (g)	Recovery (%)	Loss (%)
Labyrinthopeptin A1	0.80	0.58	72.5	27.5
Labyrinthopeptin A2	1.23	0.51	42.3	57.7

## Supplementary Materials

The following are available online at http://www.mdpi.com/2306-5354/5/2/42/s1. Figure S1: HPLC chromatogram showing the preparative HPLC separation of labyrinthopeptin A1 (**1**) and labyrinthopeptin A2 (**2**) (UV 254 nm and UV 350 nm), Figure S2: HPLC-UV/Vis chromatogram (200–600 nm) and HRMS data of **1**, Figure S3: HPLC-UV/Vis chromatogram (200–600 nm) and HRMS data of **2**, Figure S4: HPLC-UV chromatogram (220 nm) of **1** showing the purity, Figure S5: Excerpt from the original purity report of **1** (peak 3); purity expressed as area %, Figure S6: HPLC-UV chromatogram (220 nm) of **2** showing the final purity, Figure S7: Excerpt from the original purity report of **2** (peak 2); purity expressed as area %.
