# Supplementary material for: Large Scale Production and Downstream Processing of Labyrinthopeptins from the Actinobacterium Actinomadura namibiensis
Source: Bioengineering (Basel). 2018 Jun 5;5(2):42. doi: 10.3390/bioengineering5020042 (PMC6027269; doi:10.3390/bioengineering5020042)
Supplement: Supplementary file 1 [file bioengineering-05-00042-s001.pdf]

1. Preparative HPLC data

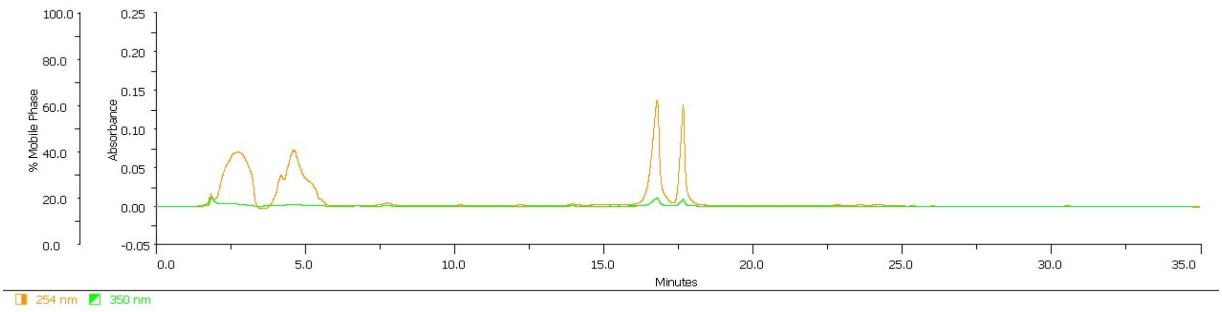

Figure S1. HPLC chromatogram showing the preparative HPLC separation of labyrinthopeptin A1(**1**) and labyrinthopeptin A2 (**2**) (UV 254 nm and UV 350 nm)

2. UHR-MS data

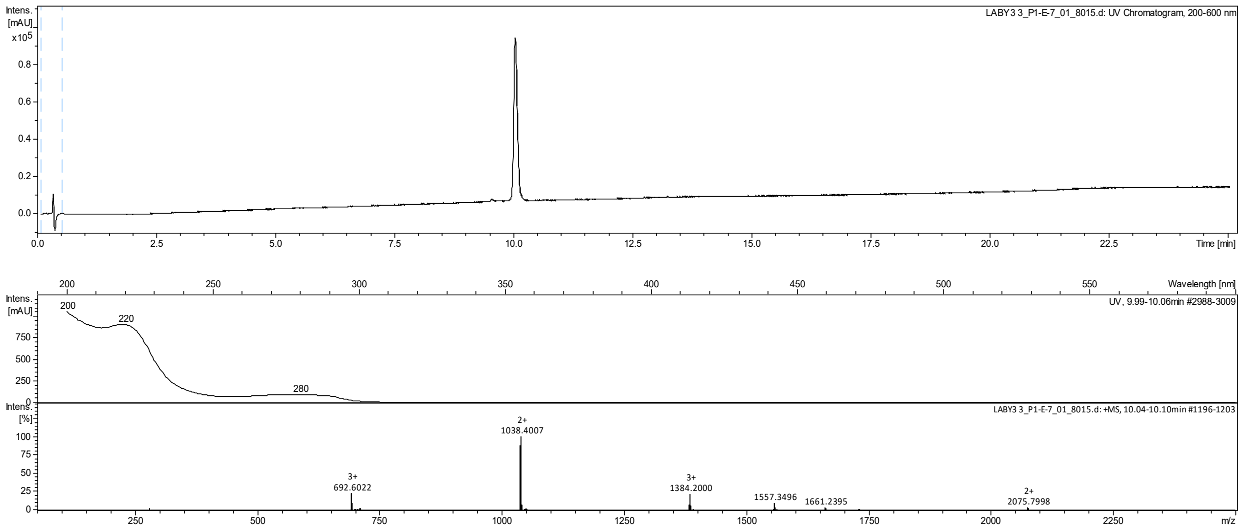

Figure S2. HPLC-UV/Vis chromatogram (200–600 nm) and HRMS data of **1**

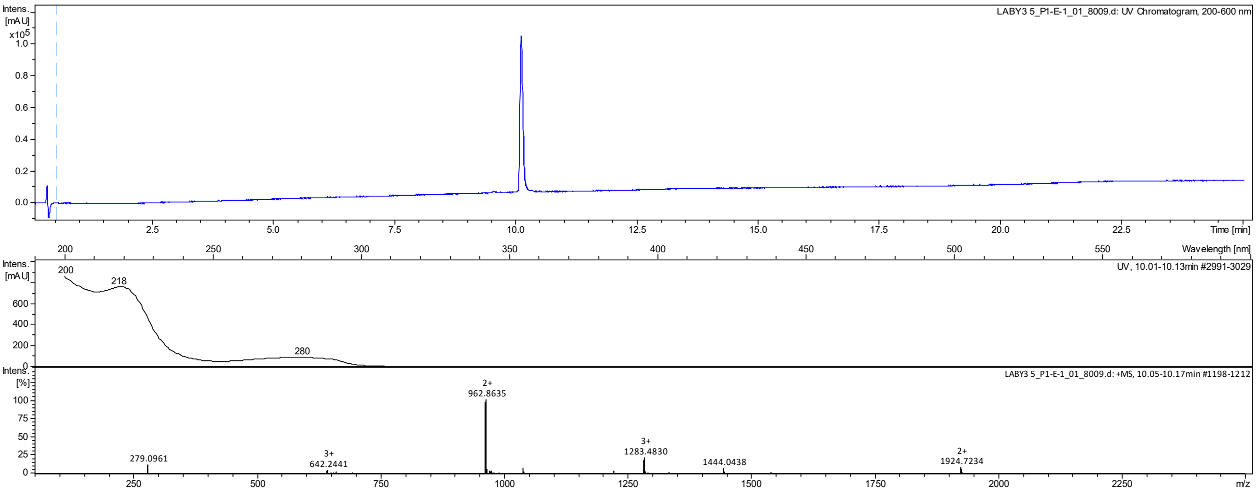

Figure S3. HPLC-UV/Vis chromatogram (200–600 nm) and HRMS data of **2**

3. HPLC data for the purity

The purity of the final samples was measured using a HPLC Agilent 1200 Series instrument (Agilent, Santa Clara, CA, USA), equipped with degasser, binary Pump SL (Agilent Technologies 1260 Infinity), autosampler and DAD/electron light scattering detector Corona Ultra RS (Dionex, Sannyvale, CA, USA). Acquity UPLC BEH C18 column (2.1 x 50 mm, 1.7 µm) from Waters (Eschborn, Germany) was used as a strationary phase while the mobile phase consisted of: solvent A- H_2O + 0.1% formic acid (FA), solvent B- acetonitrile (ACN + 0.1% FA). A gradient was run from 5% B for 5 min, followed by a linear increase to 100% B over 25 min, and thereafter maintained under isocratic conditions at 100% for 5 min; flow rate 0.6 mL/min, injection volume 2 µL, UV detection at 220 nm.

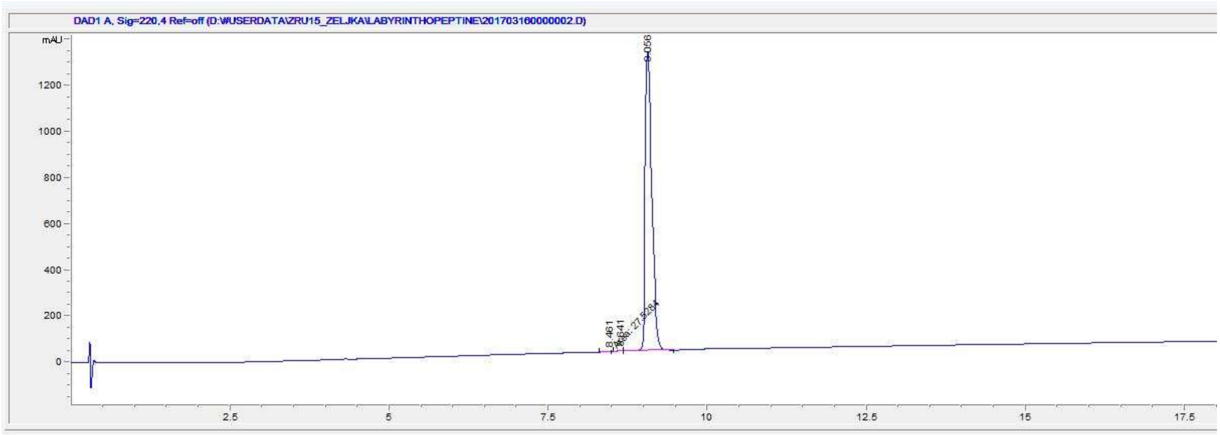

Figure S4. HPLC-UV chromatogram (220 nm) of **1** showing the purity

Signal 2: DAD1 A, Sig=220,4 Ref=off

Peak #	RetTime [min]	Type	Width [min]	Area [mAU*s]	Area %
1	8.461	MM	0.1005	27.52843	0.2930
2	8.641	VV	0.0845	29.31231	0.3120
3	9.056	VB	0.1080	9338.78809	99.3950
4	12.024		0.0000	0.00000	0.0000

Totals : 9395.62882

Figure S5. Excerpt from the original purity report of **1** (peak 3); purity expressed as area %

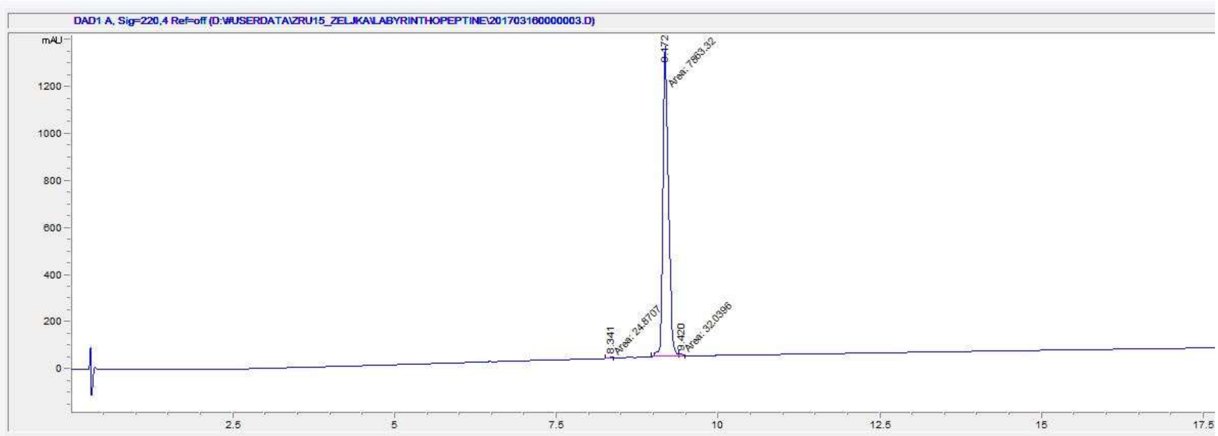

Figure S6. HPLC-UV chromatogram (220 nm) of **2** showing the final purity

Signal 2: DAD1 A, Sig=220,4 Ref=off

Peak #	RetTime [min]	Type	Width [min]	Area [mAU*s]	Area %
1	8.341	MM	0.0691	24.87069	0.3140
2	9.172	MM	0.0992	7863.32275	99.2815
3	9.420	MM	0.0642	32.03956	0.4045
4	12.024		0.0000	0.00000	0.0000

Totals : 7920.23300

Figure S7. Excerpt from the original purity report of **2** (peak 2); purity expressed as area %
